# Strategies for Therapeutic Amelioration of Aberrant Plasma Zn^2+^ Handling in Thrombotic Disease: Targeting Fatty Acid/Serum Albumin-Mediated Effects

**DOI:** 10.3390/ijms231810302

**Published:** 2022-09-07

**Authors:** Spencer Regan-Smith, Remi Fritzen, Stephen J. Hierons, Ramzi A. Ajjan, Claudia A. Blindauer, Alan J. Stewart

**Affiliations:** 1 School of Medicine, University of St Andrews, St Andrews KY16 9TF, UK; 2 Leeds Institute of Cardiovascular and Metabolic Medicine, University of Leeds, Leeds LS2 9JT, UK; 3 Department of Chemistry, University of Warwick, Coventry CV4 7AL, UK

**Keywords:** drug treatment, hypercoagulation, metabolic disease, non-esterified fatty acids, thrombosis, zinc

## Abstract

The initiation, maintenance and regulation of blood coagulation is inexorably linked to the actions of Zn^2+^ in blood plasma. Zn^2+^ interacts with a variety of haemostatic proteins in the bloodstream including fibrinogen, histidine-rich glycoprotein (HRG) and high molecular weight kininogen (HMWK) to regulate haemostasis. The availability of Zn^2+^ to bind such proteins is controlled by human serum albumin (HSA), which binds 70–85% of plasma Zn^2+^ under basal conditions. HSA also binds and transports non-esterified fatty acids (NEFAs). Upon NEFA binding, there is a change in the structure of HSA which leads to a reduction in its affinity for Zn^2+^. This enables other plasma proteins to better compete for binding of Zn^2+^. In diseases where elevated plasma NEFA concentrations are a feature, such as obesity and diabetes, there is a concurrent increase in hypercoagulability. Evidence indicates that NEFA-induced perturbation of Zn^2+^-binding by HSA may contribute to the thrombotic complications frequently observed in these pathophysiological conditions. This review highlights potential interventions, both pharmaceutical and non-pharmaceutical that may be employed to combat this dysregulation. Lifestyle and dietary changes have been shown to reduce plasma NEFA concentrations. Furthermore, drugs that influence NEFA levels such as statins and fibrates may be useful in this context. In severely obese patients, more invasive therapies such as bariatric surgery may be useful. Finally, other potential treatments such as chelation therapies, use of cholesteryl transfer protein (CETP) inhibitors, lipase inhibitors, fatty acid inhibitors and other treatments are highlighted, which with additional research and appropriate clinical trials, could prove useful in the treatment and management of thrombotic disease through amelioration of plasma Zn^2+^ dysregulation in high-risk individuals.

## 1. Introduction

Coagulation is essential for maintaining blood vessel integrity and sealing a vascular breach following injury. During this process, activated platelets adhere at the injured site via von Willebrand factor and release numerous proteins such as laminin and fibronectin as well as other molecules including Zn^2+^ ions, to enhance plug formation [1,2,3]. To strengthen the clot, a surrounding fibrin network is generated by clotting factors undergoing consecutive activation. Fibrin formation can be activated by the tissue factor protein, which is expressed by cells outside of the blood vessel (extrinsic pathway) or by internal factors present in the circulation (contact activation pathway) [4]. Fibrinolysis prevents unchecked fibrin clot growth and controls the breakdown of the fibrin network [5]. Fibrinolysis is mediated by tissue-plasminogen activator (tPA) and converts plasminogen to its activated proteolytic form, plasmin. Hypercoagulability is caused by a dysregulation of these processes, characterized by hyperactive platelets, enhanced activation of coagulation proteins and impaired fibrinolysis [6]. Hypercoagulability is heightened in disease states characterized by increased plasma fatty acid concentrations, such as obesity, type 2 diabetes and fatty liver disease, increasing the risk of occlusive vascular disease [7,8,9].

Hypercoagulability has been implicated in both arterial and venous thrombosis, key causes of mortality and morbidity worldwide [10,11,12,13]. Evidence indicates that the disruption of Zn^2+^ buffering has a direct impact on hypercoagulability [14]. Zn^2+^ acts a modulator of platelet activity and of many of the proteins involved in both the contact and extrinsic pathways of the coagulation cascade [15]. Zn^2+^ ions are released from damaged cells and platelets, increasing in concentration during tissue injury and initiating the clotting process [16]. Zn^2+^ is implicated in the activation of a wide range of proteins that regulate coagulatory pathways such as protein kinase C (PKC), fibrinogen, high molecular weight kininogen (HMWK) and histidine-rich glycoprotein (HRG) [15,17].

## 2. Zn^2+^ as a Regulator of Haemostasis

Following tissue damage, platelets mediate primary haemostasis where they adhere to the breached vascular area following their activation via an αIIbβ3 integrin-mediated process [18]. As part of this process, platelets secrete Zn^2+^, and its concentration near the surface of the platelet increases several-fold [19,20,21]. This increase in plasma [Zn^2+^] acts as a signal for platelets to adhere to the aggregate [18]. Prior to release, Zn^2+^ is stored in the platelets’ dense alpha granules, along with various proteins involved in coagulation (e.g., fibrinogen, HMWK and HRG [5]). Fibrinogen cleavage by thrombin stimulates formation of the fibrin network, the properties of which (e.g., clot density, fibre thickness) are influenced directly by Zn^2+^ [14]. HMWK and HRG aid in the neutralization of endogenous anticoagulant glycosaminoglycans (GAGs), thereby regulating coagulation [19,22]. GAGs, such as heparin, heparan sulfate and dermatan sulfate, inhibit coagulation by binding to serpins. When bound together, the GAGs can alter the conformation of the reactive loop of serpin, increasing the inhibitor activity of the molecule [22,23,24]. HMWK, HRG and fibrinogen bind and neutralise endothelial GAGs in a Zn^2+^-dependent manner. A study by Jones et al. demonstrated that the N1/N2 domain of HRG interacts with heparan sulfate and that Zn^2+^-binding to the histidine rich region (HRR) indirectly enhances cell surface binding. Furthermore, Zn^2+^ binds to fibrinogen at two different regions [25]. These are located within the D and αC domains [26]. The binding of fibrinogen to heparin has been shown to be directly influenced by the presence of Zn^2+^, with a four-fold increase in affinity upon adding Zn^2+^ [27].

The coagulation cascade can be initiated by two pathways: the intrinsic and extrinsic pathways, which act to concert fibrinogen into fibrin [5]. The intrinsic pathway, also referred to as the contact activation pathway, begins with the autoactivation of factor XII (FXII; Hageman factor), which occurs either via the association of FXII and the exposed endothelium or by interaction with inorganic polyphosphates produced from platelets [4,28]. Zn^2+^ is involved in the association of FXII with both the endothelium and organic polyphosphates, acting as an essential cofactor [28,29]. Factor XII can additionally be activated via the contact activation system, where Zn^2+^ can additionally act as a cofactor in enhancing the interaction between HMWK and FXII to endothelial cells [30]. The intrinsic pathway then proceeds with activated FXIIa converting FXI to FXIa [5]. FXIa binds to the activated platelet surface via the receptor GPIb-IX at its A3 domain in the presence of HMWK and Zn^2+^ [31]. The extrinsic pathway is activated by external trauma. Vessel injury leads to the rapid binding of platelets to the subendothelium and activation of the coagulation cascade by tissue factor (TF), exposed by trauma in the vascular wall [32]. The interaction of FVIIa and TF initiates the activation of downstream proteases, FX and FV. The binding of Zn^2+^ to FVIIa has been shown to inhibit the interaction with TF, acting as a regulator of the initiation of the extrinsic pathway [33]. Both the intrinsic and the extrinsic pathways initiate the conversion of prothrombin to thrombin, which facilitates the formation of fibrin from fibrinogen [5]. Zn^2+^ bound to fibrinogen remains bound during conversion and regulates the properties of the fibrin network [34,35]. After thrombus formation, Zn^2+^ controls the stability of the clot and its maintenance. Specifically, Zn^2+^ decreases fibrin generation time and promotes formation of thicker fibrin fibres [14,36]. Fibrinolysis is controlled by tissue-type plasminogen activator (tPA), which converts plasminogen to its active form, plasmin, also in a Zn^2+^-dependent manner [37].

**Figure 1:**
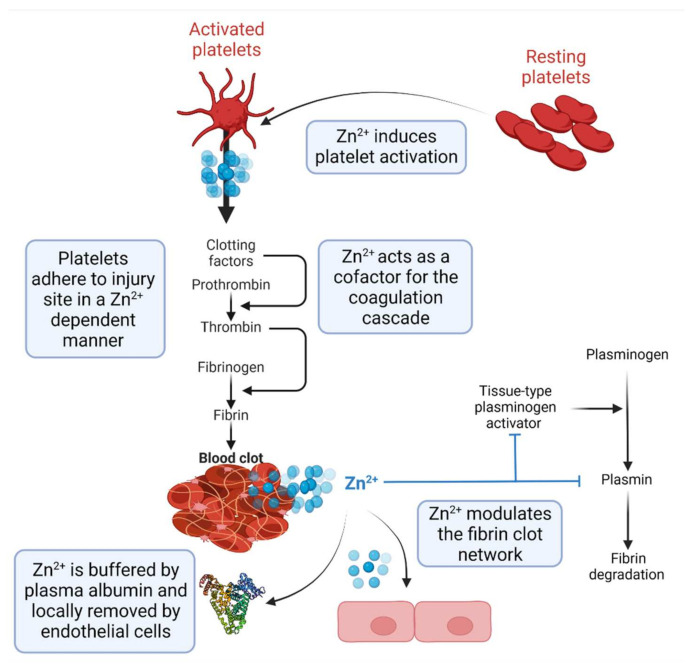
The role of Zn^2+^ in thrombus formation. Zn^2+^ acts as an essential cofactor for the activation of platelets, for the coagulation cascade culminating in the conversion of fibrinogen to fibrin and for the fibrinolytic processes that prevent vascular occlusion from thrombus breakage. Activated platelets release Zn^2+^ ions, activating surrounding resting platelets and initiating morphological changes that cause platelets to adhere to the site of the vascular breach. Zn^2+^ additionally serves as a cofactor in the initiation of both the intrinsic and extrinsic coagulation pathways that convert fibrinogen to fibrin. Finally, Zn^2+^ modulates fibrinolysis, preventing fibrin overgrowth and, ultimately, vascular occlusion. The availability of Zn^2+^ in plasma is controlled by HSA. Created using Biorender.

Given the essential roles that Zn^2+^ plays in regulating multiple aspects of coagulation, it is important that its availability in plasma is tightly controlled to prevent hypercoagulability and occurrence of thrombotic complications (Figure 1). The central role that human serum albumin (HSA) plays in this process is explained in the following section.

## 3. Zn^2+^ Binding by Human Serum Albumin and Effects of Non-Esterified Fatty Acids

**Figure 2:**
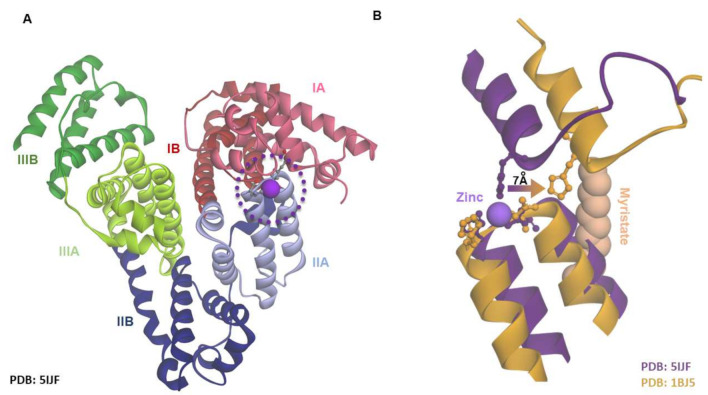
Zn^2+^ and NEFA-binding to human serum albumin. (**A**). HSA complexed with Zn^2+^ at the major Zn^2+^ binding site (often known as site A; circled) located between domains I and II and composed of His67, His247, Asp249 and a solvent water molecule. (**B**). Binding of myristate alters site A, displacing His67 by ~7 Å relative to its position in the non-fatty acid bound structure.

The total plasma Zn^2+^ concentration typically ranges from 10 to 18 µmol/L [38], the majority (75–85%) of which is bound to human serum albumin (HSA). This constitutes ~98% of the total exchangeable Zn^2+^ in plasma [5,39]. HSA also transports a multitude of other molecules and compounds, including NEFAs [40]. HSA is a single-chain protein that consists of 585 amino acids arranged into three homologous domains (I-III), each with two subdomains (A and B). A total of 17 disulfide bridges stabilize the largely alpha-helical protein structure [41]. HSA possesses a physiologically relevant Zn^2+^ binding site (site A) which is situated between domains I and II. His67 from domain I, and His247 and Asp249 from domain II and an exogenous water molecule form a distorted tetrahedral coordination sphere around Zn^2+^ (Figure 2) [39,42]. This inter-domain site displays a log *K*_D_ of −7.53 [43]. A second binding site that has a substantially lower affinity for Zn^2+^ also exists [39,44]. Chronically increased NEFAs are a common feature of several disorders, such as type 2 diabetes, obesity and fatty liver disease, that are associated with an increased risk of cerebrovascular and cardiovascular disease [14]. Plasma NEFA concentrations have been employed as a risk indicator for cardiovascular disease and sudden death [13,45]. One potential mechanism by which NEFAs contribute to the development of vascular disease may be due to their interaction with HSA and their impact on Zn^2+^ availability/speciation.

HSA possesses at least seven fatty acid-binding sites (FA1-7) of varying affinities [40,46]. Site FA2 is a high-affinity site located near to the primary Zn^2+^-binding site. Similar to the latter, it requires contributions from both domain I and II and is dependent on the convergence of two half sites formed from residues present in subdomains IA, IB and IIA [47]. Crystallographic studies have shown that upon NEFA binding to FA2, a conformational change and a significant rotation between the two domains takes place, resulting in the formation of a linear pocket. [48]. To bring about this conformational change, the bound NEFA must possess a methylene tail with 10 or more carbons [49]. This conformational change is thought to be responsible for a reduction in HSA-Zn^2+^ binding affinity. Comparison of HSA crystal structures with either Zn^2+^ or NEFAs bound suggests that binding of NEFAs causes His67 to move ~7 Å away from the other Zn^2+^-coordinating residues with a resultant reduction in Zn^2+^ affinity (Figure 2B). However, whether the binding of NEFAs at other fatty acid-binding sites on HSA impact Zn^2+^ binding has yet to be elucidated.

Given the major roles played by Zn^2+^ in haemostasis and the importance of HSA in controlling its availability, it is postulated that NEFA-lowering strategies will beneficially impact thrombotic risk in disease states. Detailed studies examining the relationship between available Zn^2+^, plasma NEFA concentration and thrombotic risk are now beginning to emerge [14]. With this in mind, we review here potential treatments and approaches that could be used to ameliorate NEFA/HSA-mediated effects on plasma Zn^2+^ handling and haemostasis.

## 4. Interventions to Ameliorate NEFA-Induced Alteration in Plasma Zn^2+^-Handling

**Figure 3:**
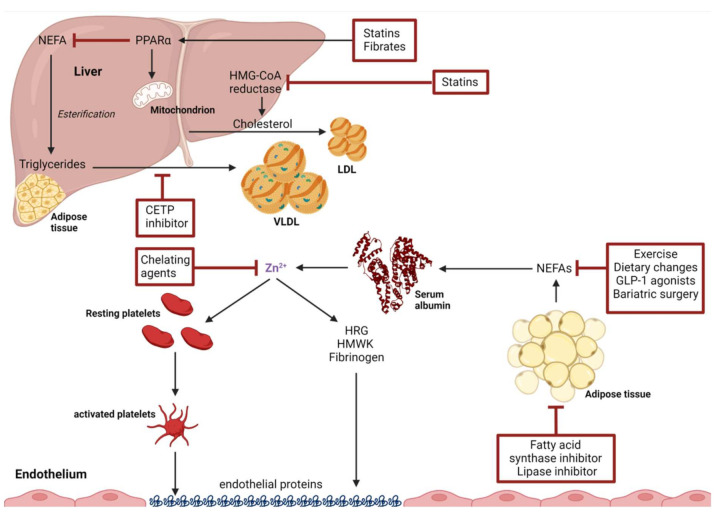
Summary of potential interventions targeting NEFA-mediated effects on plasma Zn^2+^ handling. Adipose tissue cells contain fatty acid synthase, a potential drug target and a multidomain protein that converts acyl-CoA into palmitate. Breakdown of lipids stored in the tissue by hormone-sensitive lipase and adipose triglyceride lipase allows for the release of NEFAs into the bloodstream. Such enzymes are upregulated following exercise or in response to dietary changes. NEFAs are taken up by the liver and are esterified into triglycerides. PPAR-α acts to increase mitochondrial biogenesis and inhibits triglyceride synthesis. Fibrates and, to a lesser extent, statins both target PPAR-α by stimulating its expression. Statins also inhibit the synthesis of cholesterol and the release of low-density lipoproteins (LDL) by preventing the formation of mevalonic acid, a cholesterol precursor. The action of both statins and fibrates reduces plasma NEFA levels. Triglycerides are converted into very low-density lipoproteins (VLDL) by cholesteryl ester transfer proteins (CETP), a process that can be inhibited by specific CETP inhibitors. Chelating agents may be used to reduce the availability of Zn^2+^ to bind haemostatic proteins such as HRG, HMWK and fibrinogen. Created using Biorender.

Based on current evidence, it appears likely that NEFA inhibition of Zn^2+^ binding to HSA is a contributary factor to pro-thrombotic outcomes and thrombotic risk in some disease states. Figure 3 highlights a range of different pharmaceutical and non-pharmaceutical interventions which have been shown to decrease thrombotic risk associated with a decrease in plasma NEFA or to directly decrease Zn^2+^ plasma concentrations.

### 4.1. Non-Pharmaceutical Interventions

#### 4.1.1. Exercise

An increased thrombotic risk is associated with a sedentary lifestyle and with related disorders characterised by chronically high levels of plasma NEFAs (such as obesity) [14,50,51,52]. During exercise, NEFAs are released by adipose tissue as an energy source, with their concentrations increasing two to three times compared to levels at rest [53]. NEFAs provide energy for increased heart muscle contraction, but after prolonged exercise, the body also needs to mobilise triglycerides for gluconeogenesis for the brain [50]. The associated increase in plasma NEFAs have been shown to influence Zn^2+^-HSA interactions (at least in model systems [24]), and in turn may contribute to a higher thrombotic risk observed directly after exercise [54]. The reported effects of exercise on coagulation and fibrinolysis may be due, at least in part, to an impact on plasma Zn^2+^ speciation or indeed Zn^2+^ concentration. Although plasma NEFA concentrations increase during and directly after exercise, it stands to reason that exercise may lead to decreased basal NEFA levels. The effect of a 6-week training program has been shown to decrease basal NEFA levels in slightly overweight middle-aged males (BMI 27 ± 0.5, age 50 ± 2.6). The study, although small (17 participants), shows a decrease in total plasma NEFA concentration from 0.74 mM to 0.55 mM [55]. Moreover, several studies including a systematic review by Chu et al. have examined the effect of exercise on serum Zn^2+^ concentration in athletes and non-athletes. This review showed that despite trained athletes having a higher dietary Zn^2+^ intake (+2.57 mg/day, CI: +0.97 to +4.16), their serum Zn^2+^ concentration was lower (−0.93 mol/L, CI: −1.62 to −0.23) [56]. Moreover, regular exercise has been shown to reduce age-related thrombotic risk in both men and women [57,58]. De Souza et al. compared sedentary and physically active women of pre- and post-menopausal age revealing a protective effect of exercise against an age-related hypofibrinolytic state [57]. A similar effect was observed in 23 males who underwent a 6-month physical endurance exercise program; 10 were aged 24–30 years and 13 were aged 60–82 years [58]. Significant changes in fibrinolytic variables were observed in the older group whilst no difference was observed in the younger group. A 2006 meta-analysis assessed the effect of exercise on platelet function, coagulation, and fibrinolysis [59]. The authors reported that strenuous exercise activates blood fibrinolysis and coagulation simultaneously, whilst moderate exercise enhances plasmin formation and fibrinolytic activity [54]. However, a lack of robust evidence due to the low number of participants and the lack of long-term randomised controlled trials was also acknowledged. Overall, however, exercise has a long-term positive impact on thrombotic risk, conceivably in part due to improved Zn^2+^ handling through reduction of NEFA concentrations in the resting state.

#### 4.1.2. Dietary Changes

Globalisation has increased the consumption of high levels of calorific foods, thereby driving the increase in the incidence of obesity around the world [60]. To address this issue, numerous dietary regimens have been devised, aimed at promoting weight loss (or maintaining a healthy weight). However, often little is known about the effects of these diets on plasma NEFA concentrations. A Mediterranean diet can be loosely defined as a high intake of extra virgin (cold pressed) olive oil, vegetables including leafy green vegetables, fruits, cereals, nuts and pulses/legumes, moderate intakes of fish and other meat, dairy products and red wine, and low intakes of eggs and sweets [61]. Mediterranean diets have been shown to help prevent major cardiovascular outcomes including strokes, coronary events and heart failure [62,63]. This could be partly due to an observed slight decrease in circulatory NEFA levels (−16.7% (−31.7%; −1.74%)) which correlates with an increase in adherence after 1 year of intervention [64]. This latter report by Hernáez et al. used data from the PREDIMED study (which involved 4189 Spanish participants between 2003 to 2010) to assess the usefulness of a Mediterranean diet for the prevention of cardiovascular diseases in an older population (men 55–80 years and women 60–80 years) at high cardiovascular risk (e.g., type 2 diabetes, smoking, hypertension, high concentration of LDL-C, low level of HDL-C, overweight/obese or family history of premature coronary heart disease). Overall, the study shows that the Mediterranean diet has a protective effect on cardiovascular risk [65]. Furthermore, enrichment of the Mediterranean diet with nuts lowers total NEFA levels more efficiently than enrichment with virgin olive oil compared to a low-calorie diet control group (−9.34% (−18.1%; −0.53%) and −1.62% (−10.5%; 7.32%), respectively) [65,66]. The impact of other diets on thrombotic risk has been studied; for example, a vegetarian diet has been shown to decrease thrombotic risk [67,68]. However, it is important to point out that plasma NEFA levels (or indeed Zn^2+^ plasma concentrations) are rarely reported in such nutritional studies, and it is therefore difficult to establish any causative links. In one study, Rosell et al. found the proportions of plasma long-chain n−3 fatty acids were not significantly affected by the duration of adherence to a vegetarian or vegan diet [69].

#### 4.1.3. Omega-n NEFA Supplementation

It has been shown that the unsaturated NEFA palmitoleate (C16:1) has less of an inhibitory effect on Zn^2+^ binding to albumin than its saturated equivalent, palmitate (C16:0) [14]. Whilst the inhibitory effects of any other unsaturated (or polyunsaturated) NEFAs on Zn^2+^ binding to albumin have not been examined yet in detail, the protective effects of omega-3 and -6 NEFAs for cardiovascular health have been studied since the late 1970s, with an observational study comparing a Danish population with Eskimos in Greenland [70]. This study concluded that the relative rarity of thrombotic diseases in the Eskimos was likely due to a higher level of omega-3 NEFAs in their diet, which was reflected by a higher concentration in their blood plasma.

In 2014, a study by Farsi et al. assessed the effect of supplementing the diet of diabetes patients with multiple omega-3 fatty acids in soft gel form [71]. Of 44 patients in the study, half received 4 g of omega-3 gels and the other half a placebo (corn oil soft gel) for 10 weeks. Those who took the omega-3 gels showed a significant reduction in plasma NEFA concentration between the treated arm and the placebo arm after intervention, as well as a significant reduction before and after intervention in the treated group [71]. Omega-3 supplementation was additionally found to trigger an increase in insulin sensitivity as measured by QUICKI (Quantitative Insulin Sensitivity Check Index) and HOMA-IR (Homeostatic Model Assessment of Insulin Resistance). Selvaraj et al. analysed data from the landmark REDUction of Cardiovascular Events with Icosapent ethyl intervention Trial (REDUCE-IT) which took place between 2011 and 2016 [72]. This study examined the link between icosapent ethyl, an ethyl ester of the omega-3 NEFA, eicosapentanoate (20:5), supplementation and cardiovascular risk. Approximately 8200 men and women with CVD or at high risk of developing CVD were enrolled in this study. It was reported that the treatment was efficient at reducing adverse cardiovascular outcomes, including fatal and non-fatal stroke [72]. A 2019 study by Yang et al. found that the concentrations of linoleic acid (18:2) and other omega-6 polyunsaturated fatty acids (PUFAs) are inversely proportional to the risk of cardiovascular disease. The study was undertaken in Taiwan and spanned from 1990 to 2014 with a median follow-up period of 16 years. The cohort included 1835 individuals, and by the endpoint of the study in 2014, 424 had developed CVD amongst which 244 had suffered a stroke [73]. They concluded that, in this Asian population, omega-6 PUFAs had a protective effect against CVD risks. Their data also showed that this protection was more pronounced in women. The authors did not find a link between plasma concentration of omega-3 NEFAs and CVD risk. Many n-3 NEFA supplements have been shown to positively impact on total plasma NEFAs [74], but further investigation is required to establish if and how they influence the plasma concentrations of specific NEFAs in the long term and whether and how this impacts zinc handling and its effect on haemostasis.

#### 4.1.4. Bariatric Surgery

In severely obese individuals (BMI > 40 kg/m^2^), or those with milder obesity (BMI of 35–40 kg/m^2^) together with other significant disease, bariatric surgical procedures can be recommended. Bariatric surgery is an umbrella term for several surgical procedures with the overall aim of reducing adiposity and the comorbidities accrued in severe obesity. Bariatric surgery falls under three broad types depending on their mechanism of action: (1) blocking the passage of food through the gastrointestinal (GI) tract; (2) restricting the absorption of nutrients in the stomach/intestine; (3) a combination of these mechanisms [75]. As of 2017, the two most performed procedures were sleeve gastrectomy and gastric bypass surgery, forming a total of 81.5% of the total performed bariatric surgeries worldwide [76]. Sleeve gastrectomy involves the transection of the curvature of the stomach, leading to a more rapid passage of nutrients into the duodenum. This reduces the time food is present in the stomach and decreases the surface area, ultimately reducing nutrient absorption [77]. Roux-en-Y gastric bypass (RYGB) functions by dividing the stomach into two unequally sized pouches, with the small intestine altered to attach to both [78]. Therefore, there is a restriction in the size and resultant absorption of the stomach and a blockage in the bolus passing through the GI tract. Bariatric surgery is highly effective in reducing obesity, with patients losing on average 61% of their excess weight [79]. It has been noted that there is an acute increase in NEFA concentrations post bariatric surgery due to the patient’s fasted state [80]. Long term however, bariatric surgery decreases NEFA concentrations: In 2017, Nemati et al. determined that there was a substantial decrease in NEFA concentrations, 30 months after surgery in all NEFAs apart from oleic acid [81]. This is potentially due to reduction of the triglyceride concentrations stored in the adipose tissue, depleted due to the reduced nutrients reabsorbed by the patient’s altered intestinal tract. Consequently, bariatric surgery has not only the potential to provide a long-term intervention to manage severe adiposity but could also ensure a reduction in hypercoagulability and vascular complications. Complementary to NEFA reductions, bariatric surgery has been noted to incur a reduction in plasma Zn^2+^ concentrations, with 4–9% of bariatric patients experiencing zinc deficiency preoperatively and 20–24%, 18 months after surgery [82]. Thus, there is a simultaneous reduction in both plasma NEFA and Zn^2+^ concentrations, reducing hypercoagulability. However, studies are required to measure the concentrations of NEFAs at different periods after surgery to fully support this.

## 5. Drug-Based Interventions

### 5.1. Statins

Statins are currently employed to combat atherosclerotic complications and reduce the overall concentration of cholesterol in patients at a high risk of developing cardiac disease. Statins are 3-hydroxy-3-methylglutaryl (HMG)-coenzyme A (CoA) reductase inhibitors, and primarily function by inhibiting a rate-limiting step for the synthesis of cholesterol, resulting in a reduction in the low-density lipoproteins that contain cholesterol (LDL-Cs) [83,84]. LDL-Cs transport cholesterol and other fats in the bloodstream and have been posited to be implicated in CVD in patients with elevated concentrations [85]. LDLs are primarily cleared through increased hepatic uptake. The latter is also promoted by statins, which increase hepatic LDL-C receptors [86]. Statins additionally possess other vasoprotective and antithrombotic properties, such as the improvement of endothelial function by increasing eNOS (endothelial nitric oxide synthase), stabilising atherosclerotic plaques (preventing plaque rupture and downstream vascular occlusion) and decreasing total plasma NEFA concentrations [87,88]. It is thought that the reduction in NEFA levels occurs through a combination of reduced NEFA synthesis (and subsequent release) and increased uptake by cells [89]. Statins such as simvastatin were demonstrated to decrease total NEFA concentrations by 24% over a period of 24 weeks. In addition, relative percentages of α-linoleic and linoleic acid decreased, whilst concentrations of arachidonic acid, docosahexaenoic acid and eicosapentanoic acid were unchanged [89]. Statin-induced reductions in total plasma NEFA concentrations have been demonstrated to be, in part, due to the activation of peroxisome proliferator activated receptor (PPAR)-α, a transcription factor involved in regulating the expression of genes that control fatty acid beta-oxidation and energy homeostasis, promoting the conversion of fatty acids into acetyl coenzyme A [90]. It has been demonstrated that statins activate PPAR-α via p38 MAPK and RhoA signalling pathways in adipocytes [91]. The upregulation of PPAR-α consequently increases beta oxidation in adipose (and other tissues) and reduces the availability of NEFAs in cells [92]. As the hydrolysis of triglycerides in adipose tissue releases NEFAs, it is presumed that a decrease in intracellular triglyceride concentrations ultimately results in a long-term decrease in plasma NEFA concentrations. A study by Sahebka et al. found a significant reduction in plasma NEFA levels, independent of statin type or duration of treatment [92]. Moreover, there is some evidence to indicate that statins also influence platelet aggregation, with the drug fluvastatin reducing platelet aggregation in response to arachidonic acid activation [93]. A combination of statins and fibrates is prescribed for patients with combined hyperlipidaemia, and it remains to be seen whether this improves Zn^2+^ handling, providing an added benefit towards reducing thrombotic risk [94,95]. Statins have been demonstrated to cause a significant downregulation on components of the coagulation cascade, postulated to be due to a decreased expression of TF, leading to reduced thrombin generation [87]. Colli et al. demonstrated that fluvastatin and other lipophilic vastatins inhibited TF activity in a dose-dependent manner and inhibited macrophage-mediated TF biosynthesis [96]. Elevated fibrinogen plasma levels are causative of blood hypercoagulability and increased risk of coronary artery disease (CAD). Thus, there has been significant investigation of the potential impact of statins on fibrin elevation. Undas et al. concluded that patients with elevated thrombotic risk (patients with chronic obstructive pulmonary disease (COPD)) possessed fibrin clots with denser networks that were more resistant to lysis [97]. The administration of the statin, simvastatin, improved these properties, reducing their thrombotic risk. Similar studies have confirmed these findings with fenofibrate and quinapril, which both increase clot permeability and fibrinolysis in patients’ with CAD [98]. Statins have been shown to reduce the mean serum zinc concentrations significantly, with reductions calculated to be approximately 9%, with similar changes unseen in the control patient groups [99].

### 5.2. Fibrates

Fenofibrate and related drugs are used to treat hypertriglyceridemia and other diseases with dysregulated lipid profiles [100,101]. With respect to plasma NEFA concentrations, these drugs function by activating PPAR-α, leading to an increase in NEFA oxidation and a decrease in the synthesis of triglycerides in the liver and other tissues including adipose and skeletal muscle tissues [102]. Furthermore, the activation of PPAR-α leads to a consequent increase in the cellular uptake of NEFAs by specific fatty acid transporters (FATPs) and a resultant clearance of NEFAs from blood plasma [103]. Additionally, the hypotriglyceridaemic impact of fibrates is also a consequence of impairing low-density lipoprotein (LPL) and apolipoprotein C-III (apoC-III) [104]. Due to PPAR-α-initiated repression of apoC-III synthesis, catabolism of triglyceride-rich particles such as very low-density lipoproteins (VLDLs) is enhanced, contributing to this hypotriglyceridaemic effect [102]. PPAR-α activation (as with statins) ultimately stimulates the oxidation of NEFAs in the liver, preventing triglyceride and triglyceride-rich particle synthesis, reducing NEFA concentrations in the blood plasma (through reduced synthesis/release and increased cell uptake) [105]. A meta-analysis examining different trials determined the impact of fibrates on cardiovascular outcomes, with individuals with a higher average baseline triglyceride concentration gaining a greater risk reduction for cardiovascular events [106].

Fibrates may have a beneficial impact on macro- and microvascular complications present in patients with diabetes [107]. Diabetes patients frequently suffer from microvascular issues such as nephropathy and retinopathy [108,109,110]. Damage to the micro-vessels present in the kidney can additionally lead to impaired filtration of blood plasma and loss of HSA into the urine. This heightened loss of HSA may further impair Zn^2+^ handling in plasma in a manner that is pro-thrombotic. There is some evidence that fibrates reduce kidney damage and thus reduce the loss of albumin from the bloodstream [111]. The reduction of plasma NEFAs by statins and fibrates, individually and in combination, may impair allosteric inhibition of Zn^2+^ buffering by HSA molecules. However, the contribution of this mechanism to the beneficial improvements in thrombotic risk offered by these drugs remains to be examined.

### 5.3. GLP-1 Receptor Agonists

Glucagon-like peptide 1 (GLP-1) receptor agonists are currently used to treat diabetes and promote weight loss [112]. These include the drugs dulaglutide, exenatide, semaglutide, liraglutide, and lixisenatide, which mimic the action of GLP-1 and hence stimulate insulin production. These drugs also slow the movement of food from the stomach into the small intestine, leading to patients feeling satiated faster and for longer, leading to reduced appetite and weight loss through consequent reduced calorie intake [113]. Through the metabolic changes invoked by reduced energy consumption and weight loss, these drugs have the potential to reduce plasma NEFA levels. In a trial involving 41 type 2 diabetes patients with coronary artery disease, the patients were randomized and treated with liraglutide-metformin vs. placebo-metformin during 12- + 12-week periods with a wash-out period of at least 2 weeks before and between the intervention periods [114]. It is worthy of note that most studies (on healthy individuals, individuals with type 2 diabetes or individuals with hyperlipidaemia) suggest that metformin has no effect on total plasma NEFA concentrations [74]. Fasting NEFAs were reduced in both treatment arms, but more so with liraglutide treatment (difference, −9.4 (3.9) μmol/L; *p* < 0.0001). Moreover, the plasma NEFA_nadir_, defined as the lowest concentration of NEFAs measured at any time, was lower and was reached earlier with liraglutide treatment, with a significant difference between treatments of −24.3 (0.9) μmol/L (*p* < 0.0001), where the NEFA_nadir_ decreased from 190.3 μM (at 76 min) to 149.8 μM (74 min) in the placebo group and from 187.5 μM (at 74 min) to 122.7 μM (at 68 min) in the liraglutide group [114]. In another recent trial, 62 individuals with type 2 diabetes were given either liraglutide or the sulfonylurea drug glimepiride, both in combination with metformin [115]. In total, 340 lipids and other metabolites were identified, covering 14 lipid classes, bile acids, NEFAs, amino acids and other polar metabolites. More significant changes in the metabolome following liraglutide treatment were observed compared to with glimepiride, including reduction in the plasma levels of the highly abundant NEFA stearate (mean square change after liraglutide treatment = 0.752; *p* = 0.0383). In a randomized placebo-controlled crossover study, 32 participants with normal or mildly impaired glucose tolerance received liraglutide and placebo for 3 weeks, each while on a high saturated fatty acid (HSFA) diet [116]. The HSFA diet increased plasma glucose (by 36%; all *p* < 0.01 vs. placebo). Liraglutide reduced plasma glucose by 50% and NEFA concentrations by 9% during the HSFA diet. Additionally, the HSFA diet-induced impairment of vasodilation on placebo (−9.4% vs. baseline; *p* < 0.01) was ameliorated by liraglutide (−4.8%; *p* = 0.01 vs. baseline). In this study, it was concluded that the results may be attributed to improved microvascular function and modulation of thioredoxin-interacting protein and 5′-AMP-activated protein kinase pathways in skeletal muscle. Collectively, these trials suggest that GLP-1 antagonists (potentially in combination with other drugs) may be useful in reducing plasma NEFAs to a degree that helps maintain normal plasma Zn^2+^ handling.

### 5.4. Fatty Acid Synthase Inhibitors

Fatty acid synthase (FAS) represents another potential drug target for reducing pathophysiological NEFA concentrations. FAS is present in the liver and adipose tissues and is the sole enzyme responsible for de novo synthesis of fatty acids in the body [117]. This enzyme is a homodimeric, multifunctional protein that converts malonyl CoA into palmitate [117]. Cha et al. determined that the inhibition of FAS by the novel inhibitor C75 led to concomitant expression of PPAR-α, the target of fibrates [118]. Because of this expression, there is a resultant increase in NEFA oxidation and a decrease in triglyceride synthesis in the liver [118]. FAS has also been posited as a potential target for cancer treatment due to its increased expression in many carcinomas [119]. In a drug trial, C75 was found to elicit a significant weight loss by increased fatty acid oxidation and concurrent reduced fatty acid synthesis [119]. If FAS inhibitors, such as C75, prove to be safe in clinical trials, there is the potential for these to be repurposed to reduce plasma NEFA levels in obese patients and those with type 2 diabetes. As of this publication, there are no reports conducted to assess the impact of FAS inhibitors on thrombotic risk in patients. However, there is a clear necessity for future research to evaluate potential benefits, especially given the fact that the plasma NEFAs derived from de novo lipogenesis (especially the saturated NEFAs palmitate and stearate) are the most elevated in obese and type 2 diabetes patients [74], and the majority of these are highly efficient at impeding Zn^2+^-binding to HSA [14].

### 5.5. Lipase Inhibitors

There are two major routes of plasma NEFA generation with the contribution of each pathway depending largely on the nutritional status of the individual. In the fasted state, the majority of plasma NEFAs (to be bound by albumin) are derived from the intracellular hydrolysis of triglycerides (TG) in adipose tissue (endogenous NEFAs) [120]. The liberation of NEFAs from these fat stores is catalysed by adipose triglyceride lipase (ATGL) and hormone-sensitive lipase (HSL) [121]. Specific inhibition of these lipolytic enzymes as a means of lowering plasma NEFAs would not be a suitable therapeutic strategy, as any potential CV benefit incurred by NEFA lowering would be heavily outweighed by the metabolic and systemic abnormalities that result from the lower rates of degradation of cytoplasmic triglycerides (as is evidenced in individuals with hereditary deficiency of AGTL or HSL [122]). NEFAs are also required for cardiac muscle contraction [58] and for gluco-neogenesis. The second major pathway of plasma NEFA generation occurs after eating a fat-containing meal (exogenous NEFAs). Here, dietary TGs, packaged in lipid-rich particles known as chylomicrons, are hydrolysed in the capillaries of adipose tissue and other tissue fluids by lipoprotein lipase (LPL). Most of the LPL-generated NEFAs are taken up and stored by adipocytes, but a proportion of “spillover” occurs and enters the systemic circulation [123]. It has been shown that these spillover NEFAs can make a considerable contribution (40–50%) to the total plasma NEFA pool in the post-prandial period [124]. Reducing the proportion of spillover-derived NEFAs perhaps represents a more suitable strategy of NEFA management. Orlistat (Xenical) is a prescribed weight loss aid and may target this pathway indirectly. Orlistat is a lipase inhibitor which is taken after eating a fat-containing meal. The drug reduces rates of TG hydrolysis in the gastrointestinal tract and thus reduces the fraction of NEFAs and mono-acylglycerol absorbed by enterocytes [125,126]. Overall, this would restrict the formation and release of TG-containing chylomicrons from enterocytes and subsequently lower chylomicron-TG hydrolysis in the adipose capillary. The drug has been shown to positively impact the lipid profile in at-risk individuals including reductions in plasma NEFA levels [127,128]. Orlistat has also been shown to inhibit the thioesterase domain of fatty acid synthase (FAS) (discussed above), an activity that may contribute to its NEFA-lowering properties [129]. Recently, the effect of long-term orlistat administration on cardiovascular outcomes in obese individuals was tested in a large propensity-score matched cohort study. Individuals who had taken orlistat as part of their treatment regimen were less likely to suffer from a major-adverse cardiovascular event compared to those who had not taken orlistat therapy. Whether this is in part due to an improvement in Zn^2+^ handling remains to be determined.

### 5.6. Cholesteryl Ester Transfer Protein Inhibitors (CETP Inhibitors)

CETP is a glycoprotein synthesized in the liver and secreted in the plasma, where it plays a critical role in lipoprotein particle homeostasis [130]. CETP transports triglycerides and cholesteryl esters between lipoproteins. Cholesteryl ester is transported from HDL to VLDL and LDL in a CETP-dependent manner, whilst TGs are transported from VLDL and LDL to HDL until equilibrium is reached. Four CETP inhibitors have been developed and shown to induce an increase in HDL cholesterol concentration (torcetrapib, dalcetrapib, evacetrapib and anacetrapib). Of these, only anacetrapib led to a significant decrease in cardiovascular risk. The other three were abandoned during their respective phase-III trials, as the beneficial effects were considered too low to pursue further development [131]. The effect of anacetrapib on plasma NEFA levels is not known. However, torcetrapib has been shown to reduce plasma NEFA concentration by 23% in an insulin-resistant and dyslipidaemia hamster model [132]. A fifth CETP inhibitor, TA-8995, is currently being developed after a successful phase II clinical trial. Overall, the effect of CETP inhibitors on Zn^2+^-dependent thrombosis has not been assessed but could be examined in future trials.

## 6. Zn^2+^-Targeted Interventions

### 6.1. Chelation Agents

Chelation-based drugs have long been suggested as a potential treatment for the thrombotic complications present in obesity and diabetes [133]. This treatment has been recommended by clinicians as a method for increasing blood flow in diseased and clotted blood vessels, without the need for surgical intervention [133]. The basis for this approach is primarily to sequester Ca^2+^, but such agents are also likely to influence the speciation of plasma Zn^2+^. The treatment involves administering compounds into the bloodstream to form stable, water-soluble metal complexes. Thus, theoretically, complexed Zn^2+^ could be prevented from binding to proteins such as HRG or HMWK and initiate coagulation processes. Currently, chelating agents such as 2,3-dimercaprol and EDTA are employed in clinical settings to counter the effects of heavy metal poisoning from compounds such as lead and arsenic [134]. Although these treatments successfully sequester clinically toxic concentrations of heavy metals, side effects such as fever, headache and nausea are common [134]. Due to their lack of specificity, such chelating agents will affect the speciation of Zn^2+^, but also other essential trace metal ions such as Ca^2+^, Cu^2+^ and Mg^2+^, thereby requiring additional supplementation and careful monitoring in tandem.

Regarding chelation therapy in the context of cardiovascular disease, a double blind, placebo-controlled, randomized trial enrolled 1708 patients with a history of myocardial infarction and administered the participants with 40 infusions of either an ethylenediaminetetraacetic acid (EDTA)-containing chelation solution or an ascorbate placebo [135]. The trial primary end point was a composite of fatal and non-fatal outcomes. From the results presented, there seemed to be no significant difference between the two groups in terms of fatal and non-fatal outcomes. However, two subgroups appeared to benefit from the treatment, namely patients with a prior history of anterior myocardial infarctions (MI) and patients with type 2 diabetes. This latter subgroup was further assessed in a subsequent publication [136]. Note that the dissociation constant of Zn^2+^-EDTA complexation (log *K*_D_ = −16.5) is not only much lower than that for Ca^2+^-EDTA (log *K*_D_ = −10.65) [137], but is also nine orders of magnitude lower than that for the Zn^2+^-HSA complex. This suggests that a significant proportion of Zn^2+^ in plasma would be chelated by EDTA even though the total plasma concentration of Zn^2+^ is around two orders of magnitude lower than that of Ca^2+^ (tens of micromolar compared to single unit millimolar, respectively). A second trial (TACT2) has been designed to address specifically the question of chelation treatment efficacy in post MI and type 2 diabetes patients. TACT2 is yet to be completed, with an estimated completion date at the end of 2023. Chelation therapies have also been shown to increase the efficacy of tissue plasminogen activator (tPA, alteplase), the only treatment available for acute thrombotic complications such as strokes, pulmonary emboli and myocardial infarctions. An in vitro and in vivo study has shown that zinc (and iron) metal ions appear to have an inhibitory effect on fibrinolysis, with their removal via chelation reducing their concentrations and thus their ability to inhibit tPA mediated thrombolysis [36,138]. However, the mechanism by which zinc (and iron) act on tPA is currently unknown.

### 6.2. Targeting the FA2 Site of HSA

In the future, inhibitors could be designed to specifically prevent the allosteric switch that occurs when HSA binds to NEFAs, thereby preventing the consequential Zn^2+^ mishandling via NEFA inhibition. Shorter chain NEFAs such as octanoate (or derivatives) may bind at FA2 but are considered too short to invoke the allosteric structural change that influences Zn^2+^ binding [14,139]. Recently, however, lipoic acid ((*R*)-5-(1,2-dithiolan-3-yl)pentanoic acid, which also has an eight-carbon chain) was demonstrated to restore Zn^2+^ binding to albumin in the presence of NEFAs [140]. Although the molecular mechanism for this effect remains unknown, it may be speculated that lipoic acid could bind to the FA2 site more strongly than the latter but due to its shortness without eliciting the conformational change. Other potential candidates for this or similar mechanisms are the cannabinoid derivatives URB597 and AM5206 which have both been predicted to bind to sites close to the FA2 site on HSA. However, their ability to inhibit NEFA binding at FA2, whilst preserving the ability of HSA to bind Zn^2+^, has yet to be assessed [141]. Regardless of the usefulness of shorter chain NEFAs or cannabinoids in this context, it remains possible that molecules that bind to HSA and prevent the allosteric switch may be identified or designed. However, significant challenges would remain, as a sufficient proportion of the plasma HSA molecules (ca. 42 mg/mL; [142]) would require to be inhibited to prevent alterations in plasma zinc buffering induced by chronically high NEFA levels.

### 6.3. Downstream Targets of Disrupted Zinc Buffering

Beyond albumin, downstream targets such as coagulatory proteins and platelet receptors could be utilised to prevent Zn^2+^-mediated hypercoagulation. Complement 3 protein (C3) cross-links into the fibrin network, induces a resistance to fibrinolysis and reduces the fibrinolytic rate [143]. An affimer designed by King et al. inhibited the interaction between C3b and fibrinogen and has been posited as a potential future novel treatment [144]. An affimer such as this could mitigate some of the thrombotic risk posed by disrupted zinc buffering in patients with pathophysiological concentrations of NEFAs. In addition, αIIbβ3 integrins are present on the surface of the platelet and bind fibrinogen at the RGD site in a Zn^2+^-dependent manner [145]. A small molecule inhibitor screen (involving 33,264 compounds) identified a 1,2-pyrimidine derivative that inhibited fibrinogen binding. Additional downstream targets such as HRG and HMWK could also be targeted by affirmers, nanobodies or small molecule inhibitors to prevent the potential downstream hypercoagulation caused by the increased proportion of available Zn^2+^. Any future inhibitor would require a strict and tightly controlled dosage for the patient to compensate for the relative increase in available Zn^2+^ in the patient’s plasma.

## 7. Conclusions

This review has highlighted the potential role of NEFAs in allosteric regulation of Zn^2+^ buffering by HSA and consequent effects on thrombosis risk. Moreover, a range of potential treatments and preventative measures to reduce the concentrations of NEFAs (or their effect on Zn^2+^ handling) in plasma are presented. We acknowledge that the treatments examined have pleiotropic effects, but it is possible that some may be attributable to the NEFA–HSA–Zn axis. Longer and more specialized studies are required to fully validate the benefits of the highlighted treatments on the reducing of plasma NEFA concentrations and the impact these have on plasma Zn^2+^ dysregulation (including speciation) and haemostatic processes. Clinicians assessing thrombotic risk should consider NEFA concentrations and their potential effects, in addition to other lipid markers. This review recommends further studies centred around the highlighted interventions. Due to their recent discovery and development, lipase and fatty acid synthase inhibitors must be further evaluated to ascertain their effectiveness at reducing thrombotic risk in patients with hypercoagulability. A definitive conclusion from trials such as TACT2 is required before the clinical implementation of chelation therapies. It is also possible that the interventions highlighted may have a greater effect in combination, potentially acting to reduce NEFA levels and chelate the increased proportion of available Zn^2+^ simultaneously. NEFA-mediated Zn^2+^ dysregulation is likely to be an important mechanism by which thrombotic risk is increased, particularly as Zn^2+^ is known in vitro to impact upon haemostasis through multiple mechanisms, which include regulation of platelet function, fibrin clot formation and fibrinolysis. A fuller understanding of these effects and how these could be targeted therapeutically could be used to better treat and manage such complications in high-risk individuals.
